# Tailoring Peptide Self-Assembly and Formation of 2D Nanoribbons on Mica and HOPG Surface

**DOI:** 10.3390/ma15010310

**Published:** 2022-01-02

**Authors:** Hao Kong, Bin Liu, Guozheng Yang, Yun Chen, Gang Wei

**Affiliations:** College of Chemistry and Chemical Engineering, Qingdao University, Qingdao 266071, China; konghao5@outlook.com (H.K.); liubinname@outlook.com (B.L.); yangguozheng123@outlook.com (G.Y.); chenyunwu823@outlook.com (Y.C.)

**Keywords:** self-assembly, peptide, nanofibers, nanobelts, surface, materials science

## Abstract

Studying the interactions between biomolecules and material interfaces play a crucial role in the designing and synthesizing of functional bionanomaterials with tailored structure and function. Previously, a lot of studies were performed on the self-assembly of peptides in solution through internal and external stimulations, which mediated the creation of peptide nanostructures from zero-dimension to three-dimension. In this study, we demonstrate the self-assembly behavior of the GNNQQNY peptide on the surface of mica and highly oriented pyrolytic graphite through tailoring the self-assembly conditions. Various factors, such as the type of dissolvent, peptide concentration, pH value, and evaporation period on the formation of peptide nanofibers and nanoribbons with single- and bi-directional arrays are investigated. It is found that the creation of peptide nanoribbons on both mica and HOPG can be achieved effectively through adjusting and optimizing the experimental parameters. Based on the obtained results, the self-assembly and formation mechanisms of peptide nanoribbons on both material interfaces are discussed. It is expected that the findings obtained in this study will inspire the design of motif-specific peptides with high binding affinity towards materials and mediate the green synthesis of peptide-based bionanomaterials with unique function and application potential.

## 1. Introduction

Peptides are a kind of natural macromolecular compound composed of amino acids, which have exhibited various functions and bioactivities, showing wide applications in various fields [1]. Therefore, the design and synthesis of peptide-based nanomaterials have attracted great interest in the last decades. Previously, more focus was on the folding, unfolding, and aggregation mechanism of proteins and peptides that related to human diseases, such as Alzheimer’s disease, Parkinson’s disease, Huntington’s disease, and type II diabetes [2,3,4]. With the development of nanotechnology and materials science, it has been realized that the aggregated peptide/protein nanostructures are excellent precursors or templates for the synthesis of functional nanomaterials.

Nanomaterials formed by self-assembly of peptides have superior and unique physical and chemical properties, such as high mechanical strength, high thermal stability, high biocompatibility, simple preparation, and customizable functions [5,6,7]. These excellent properties provide peptides with a wide range of biological applications, such as biomedicine, bioimaging, photothermal diagnosis and treatment, biosensing, tissue engineering, and other applications [8,9,10,11,12,13].

In the process of peptide self-assembly, the formation of nanostructures is affected by factors such as pH, temperature, molecular concentration, ionic strength, and organic stimulants [14,15]. Therefore, it is possible to obtain nanostructures with different dimensions from monomer to one-dimensional (1D), two-dimensional (2D), and three-dimensional (3D) structure by adjusting appropriate conditions [16,17,18]. Peptides can not only self-assemble into excellent nanostructures in solution and on the interface, but also self-assemble on the surface of inorganic templates (such as mica, highly oriented pyrolytic graphite (HOPG), MoS_2_, and other substrates). Mica and HOPG are commonly used substrate materials for AFM because of their smooth, clean, and renewable surface. The mica used for AFM is generally muscovite; the structural formula is K_2_O·Al_2_O_3_·SiO_2_, the surface is negatively charged, and the hydrophilic mica can be modified to become a hydrophobic surface [19]. HOPG is a relatively new form of high-purity carbon, and the freshly uncovered surface is composed of many atomic layers of steps. Compared with mica, HOPG has no polarity at all and has good electrical conductivity [20]. Furthermore, peptides have been proven to be able to combine with a variety of functional nanomaterials, such as graphene [21], metal organic frameworks [22], MXene [23], cellulose [24], etc., due to their tunable surface groups. Peptides are often effectively combined with other functional materials through interactions such as hydrogen bonds, electrostatic interactions, and hydrophilic/hydrophobic interactions [25].

Previously, Zhang et al. found that the peptide Gav-9 (NH_2_-VGAVVAGV-CONH_2_) self-assembled on the surface of hydrophilic mica and hydrophobic HOPG into nanowires with triple symmetric orientation [26]. GAV-9 nanowires are horizontally oriented on HOPG and “upright” oriented on mica. Chen et al. prepared ordered 2D arrays and films on MoS_2_ and HOPG with specific peptide YSATFTY, and they further studied the formation mechanism of ordered 2D arrays through in-situ AFM and molecular simulation [27].

GNNQNY is a heptapeptide derived from the yeast prion protein Sup35 and was selected as the “Eisenberg family” [28]. In previous studies, it was found that they were mainly incubated into nanofibers and nanocrystals [29]. As a classic peptide sequence, people mainly focused on its fiber aggregation mechanism [30].

Here, we studied the ordered self-assembly of the sequence peptide GNNQQNY derived from prion protein on the surface of hydrophilic mica and hydrophobic HOPG. GNNQQNY peptides self-assemble on the surface of mica to form directional nanoribbons or nanofiber structures with an angle of 60 degrees, mainly bidirectional nanoribbons, and their self-assembly is affected by factors such as solvent, pH, concentration, and evaporation time. Different from self-assembly on the surface of mica, GNNQQNY peptides are easier to self-assemble on the surface of HOPG to form 2D nanoribbon structures with an angle of 60 degrees. While exploring the influencing factors of the self-assembly of GNNQQNY on the surface of mica and HOPG, we obtained its formation mechanism, that is, the peptides are affected by the substrate lattice and undergo one-dimensional orderly epitaxial growth on the surface, and they are connected laterally to form nanobelts.

## 2. Materials and Methods

### 2.1. Materials and Reagents

The peptide (purity 95%) with a sequence of GNNQQNY was bought from the SynPeptide Biotechnology Co., Ltd. (Nanjing, China). Mica was purchased from the XingDongFu electronic Co., Ltd. (Guangzhou, China), and HOPG (7 mm × 7 mm × 1 mm, Grade ZYB) was purchased from the Structure Probe, Inc. (West Chester, PA, USA). Trifluoroacetic acid (TFA, 99%), trifluoroethanol (TFE, 99.5%), HCl (36–38%), and NaOH (96%) were provided by the Sinopharm Chemical Reagent Co., Ltd. (Beijing, China). All chemicals used in this work were analytical reagent grade and directly used without additional purification. Ultrapure water used in the whole experiment was produced from a Millipore system (≈18.2 MΩ cm^−1^).

### 2.2. Self-Assembly of Peptide on Mica and HOPG Substrates

Peptide powder was solved with a solvent (such as ultrapure water) to prepare peptide solutions with a concentration of 0.5 mg/mL. Then, the peptide solution was diluted with corresponding solvent to a concentration of 0.25 mg/mL. After that, 10 μL peptide solution was dropped onto the freshly cleaved mica or HOPG surface, letting the peptide molecules self-assemble into various nanostructures. In order to mediate the self-assembly of peptide, the mica or HOPG with dropped peptide solution was kept in a closed sample box to reduce the evaporation velocity of liquid. The environmental temperature was 20 °C, and the relative humidity was about 50%.

### 2.3. Tailoring the Self-Assembly of Peptide under Various Conditions

*Solvent effect:* Five solvent systems, such as ultrapure water, 0.1% FEA, TFE, water/TFE (*v/v*, 1:1), and 0.1% TFA/TFE (*v/v*, 1:1) were used to prepare and dilute the peptide samples.

*Peptide concentration effect*: Different peptide concentrations, such as 0.1, 0.2, 0.25, 0.3, 0.4, and 0.5 mg/mL were utilized.

*pH value effect*: To check the self-assembly of peptide on acidic, neutral, and basic solutions, the pH of the peptide solution was adjusted to 2, 7, and 12, respectively.

*Evaporation velocity effect*: The closed and open sample box were used for adjusting the evaporation velocity of liquid.

### 2.4. AFM Characterizations of Self-Assembled Peptide Nanofibers and Nanoribbons

All atomic force microscopy (AFM) samples were prepared by dropping 10 μL peptide samples onto the freshly cleaved mica or HOPG substrates and dried in a closed or open sample box in air for characterization. AFM measurements were performed in air using the FM-Nanoview 6800 AFM (FSM-Precision, Suzhou Flying Man Precision Instrument Co., Ltd., Suzhou China) with tapping mode. Silicon probes of type Tap300Al-G (300 kHz, 40 N/m) were used for AFM image capturing.

### 2.5. Statistical Analysis

The tapping mode images and corresponding section analysis, as well as 3D morphology, were carried out with Gwyddion software (Version 2.57). The obtained data were analyzed with Origin software (Version 2021) for statistical analysis on the height, width, and length of self-assembled peptide nanostructures.

## 3. Results and Discussion

**Scheme 1:**
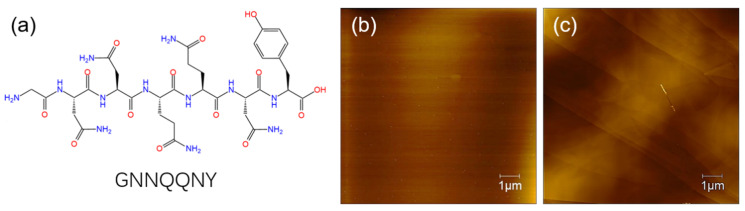
(**a**) Molecular structure of GNNQQNY peptide; (**b**) AFM image of freshly uncovered mica; (**c**) AFM image of freshly uncovered HOPG.3.1. Self-assembly of GNNQQNY on mica surface.

In this section, we studied the self-assembly of GNNQQNY peptide on the surface of both mica and HOPG using AFM characterization. The molecular structure of the GNNQQNY peptide is shown in Scheme 1a, and then Scheme 1b,c prove that the freshly uncovered mica and HOPG are clean. The formation of peptide nanofiber and nanoribbon arrays with fine arrangement is analyzed and discussed.

**Figure 1:**
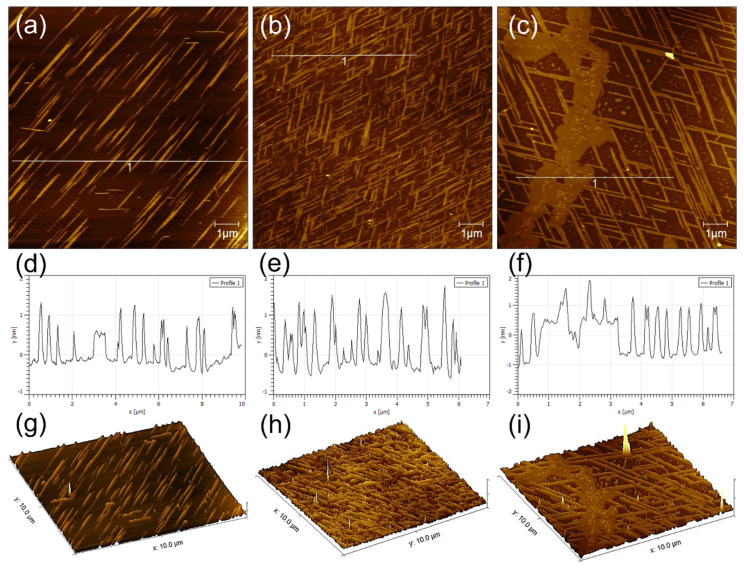
AFM characterizations of self-assembled (**a**,**d**,**g**) mono-directional, (**b**,**e**,**h**) bi-directional, and (**c**,**f**,**i**) tri-directional peptide nanobelts: (**a**–**c**) height images, (**d**–**f**) section analysis, and (**g**–**h**) 3D morphologies.

GNNQQNY peptides self-assemble on the mica surface to form nanofibers and nanoribbons with the height of 0.6–1.5 nm and nanoribbons with the height of 1.5–2.2 nm. In addition, the peptides also form some thin films on the mica surface. As shown in Figure 1a–c, the formed nanostructures show highly regular directionality among each other, and several distribution patterns often appear mainly including single directional nanoribbons, bi-directional nanoribbons with an intersection angle of 60°, and tri-directional nanoribbons with an intersection angle of 60°. Among them, bi-directional nanoribbons with an intersection angle of 60° are the most common, and unidirectional and tri-directional nanoribbons are not easily formed. As shown in Figure 1d–f, the section analysis corresponding to the AFM height images of Figure 1a–c indicates that these nanoribbons/nanofibers are highly uniformly distributed. Figure 1g–i shows a 3D morphological analysis that corresponds to the AFM images in Figure 1a–c, which clearly proves the formation of directional structure of GNNQQNY peptides on mica.

**Figure 2:**
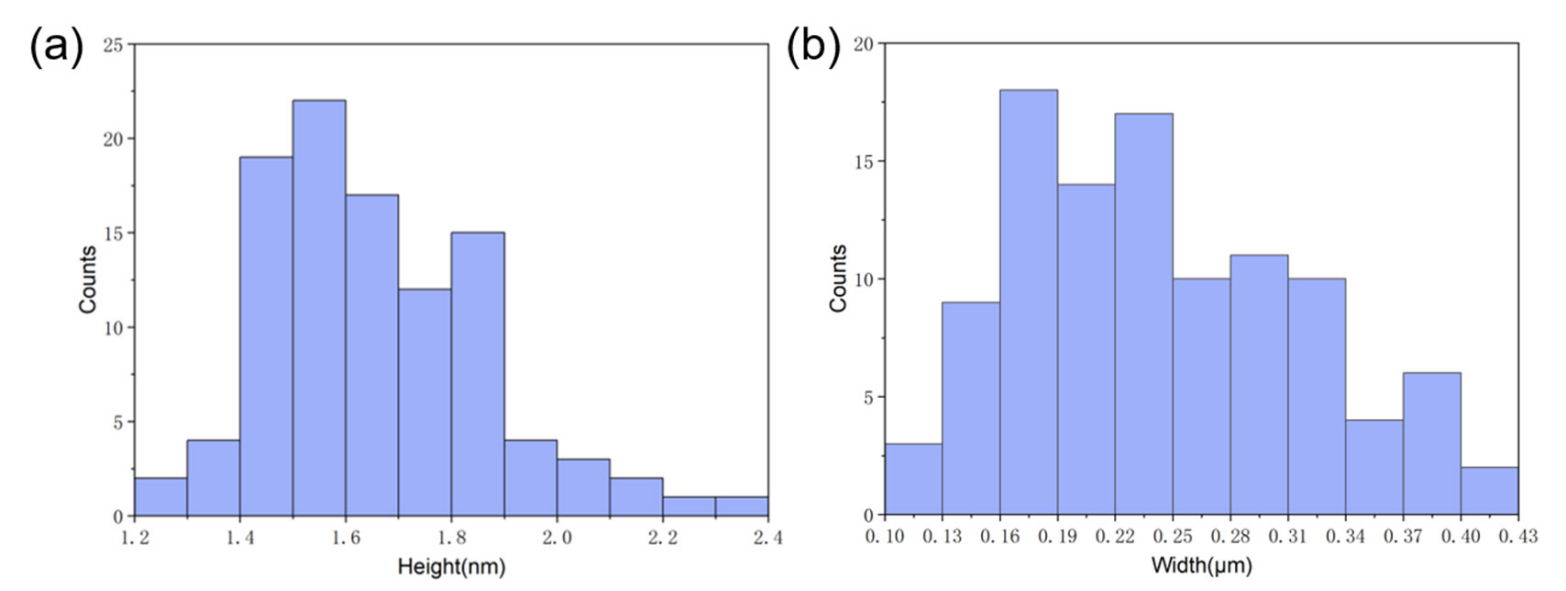
The statistical height (**a**) and width (**b**) analysis of self-assembled peptide nanobelts that synthesized in mixed (*v/v*, 1:1) TFA (0.1%) and TFE.

To investigate the dimensions of the nanoribbons that were formed by the self-assembly of GNNQQNY on the mica surface, we performed the statistical analysis of the heights and width of peptide nanoribbons that are shown in Figure 1b, and the histogram distributions are shown in Figure 2. It can be found that the height of peptide nanoribbons is mainly concentrated in the interval of 1.4–1.9 nm, with the average height at 1.65 nm (Figure 2a). In addition, the width of peptide nanoribbons is mainly concentrated in the interval of 160–340 nm, with the average width at 240 nm (Figure 2b). It should be noted that the height of the self-assembled peptide nanoribbons is uniform in all the obtained AFM images, while the width of nanoribbons varies with the change of experimental conditions.

### 3.1. The Effect of Organic Solvents on the Self-Assembly of Peptide

Polar organic solvents can promote the self-assembly of peptide molecules in solution and on the surface of mica by affecting non-covalent interactions between molecules. To explore the effects of organic stimulants on the self-assembly of GNNQQNY peptides on mica surface, several solvent systems were used to test their effects.

**Figure 3:**
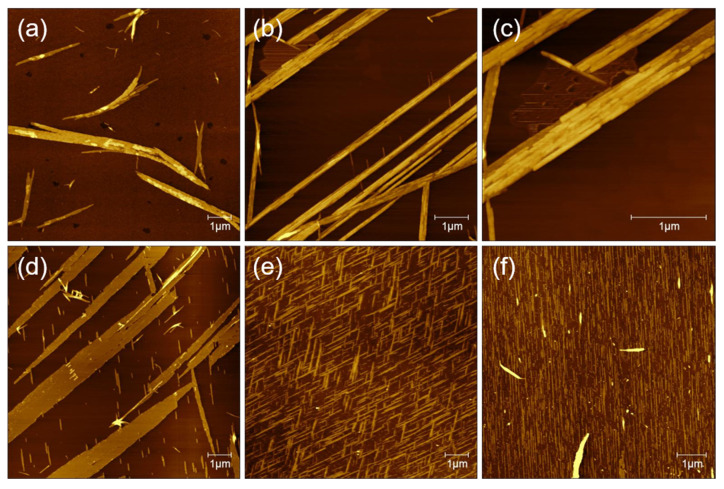
The effects of various solution systems on the structure of peptide nanobelts: (**a**) water, (**b**,**c**) TFE, (**d**) 0.1% TFA, (**e**) 0.1%TFA/TFE (*v/v*, 1:1), and (**f**) water/TFE (1:1).

As shown in Figure 3a, in pure water solvents, the AFM image indicates that GNNQQNY peptides form mainly irregular nanofibers or nanoribbons, which is consistent with previous reports on the formation of GNNQQNY nanostructures in pure water [28]. In the TFE solution, it is found that straight peptide nanoribbon structure is arranged in a single direction, and at the edge of the nanoribbons some small nanofiber structures are created. It is interesting that the formed nanofibers are parallel in the other direction, indicating that the self-assembly of peptide molecules on the surface of mica forms a single direction nanoribbon structure at first and then begins to form directional nanostructure with an angle of 60 degrees (Figure 3b).

To see the detailed structure of bidirectional peptide nanoribbons, peptide nanoribbons are magnified, and the zoomed AFM height image is shown in Figure 3c. It can be seen that the nanoribbons are formed by a horizontal connection of nanofibers and the double-layer nanoribbon structure is formed. The height of the single-layer nanoribbons is about 2 nm, and the height of the double-layer nanoribbons is about 4 nm. Meanwhile, the height of the formed nanofibers at the edge of the nanoribbons is about 1 nm, which indicates that the nanoribbon structure is not only formed by the horizontal connection between the nanofibers, but also the vertical stacking of a few layers of nanoribbons. The AFM image of GNNQQNY dissolved with TFA (0.1%) and incubated on the mica surface is similar to that dissolved with a TFE solution, with a single direction of wide nanoribbons that accompanied by a thin nanofiber in the other direction (Figure 3d). The difference is that the nanoribbons formed with TFA (0.1%) show a width of up to 1.12 μm at their widest point, and the height of the formed nanoribbons is only about 1.5 nm. 

When the solution system was changed to the mixture (*v/v*, 1:1) of TFA (0.1%) and TFE, the obtained AFM image (Figure 3e) indicates that dense bidirectional nanoribbons and thin nanofibers are created, which arrange in the same direction. Similarly, the peptide dissolved in a mixture (*v/v*, 1:1) of water and TFE also induced the formation of peptide nanoribbons in one direction on mica surface, as shown in Figure 3f.

Based on these AFM data, we suggest that the addition of organic polar solvents to the peptide solution system promotes the formation of peptide nanoribbons/nanofibers with directional arrays, and different organic polarity solvents cause specific effects on the self-assembly and conformation transition of GNNQQNY peptides [14]. However, water, which is also a polar solvent, cannot affect the conformation transition of the GNNQQNY peptide.

### 3.2. The Effect of Peptide Concentration Is on Their Self-Assembly

**Figure 4:**
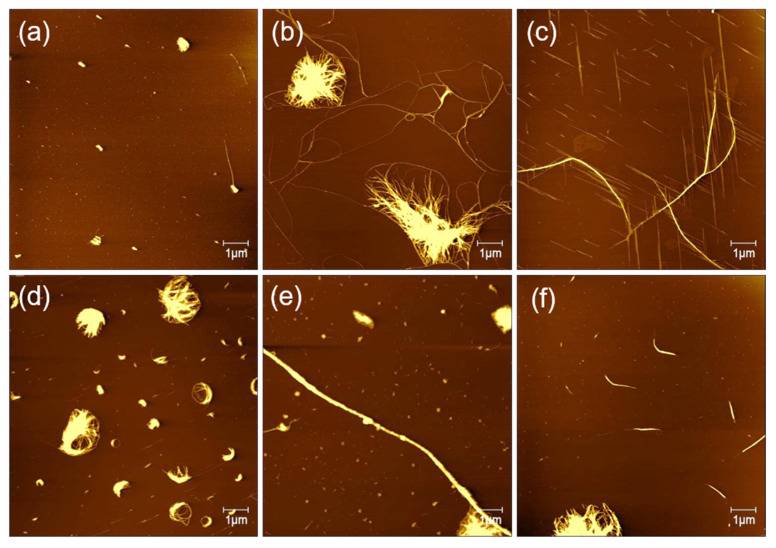
Self-assembled peptide nanostructures on mica by adjusting the peptide concentration with (**a**) 0.1, (**b**) 0.2, (**c**) 0.25, (**d**) 0.3, (**e**) 0.4, and (**f**) 0.5 mg/mL.

The solution concentration is decisive for the incubation of peptides on the surface of mica to form directional nanoribbons and nanofibers. Figure 4 shows AFM images of different concentrations of peptides that incubated on mica for surface-guided molecular self-assembly. When the peptide concentration was of 0.1, 0.2, 0.3, 0.4, and 0.5 mg/mL, the fiber clusters were observed on mica, and no directional filament or nanoribbon structure was observed. At the concentration of 0.25 mg/mL, it can be observed that not only curved long fibers, but also directional nanofiber and nanoribbon structures were formed on the surface of mica. According to previous report [28], the peptide GNNQQNY in aqueous solution will self-assemble to form fibers immediately. We believe the peptide concentration of 0.25 mg/mL is optimal for inducing the self-assembly and formation of directional nanofibers and nanoribbons on mica surface. 

### 3.3. The Effect of pH on Peptide Self-Assembly

The pH of solution system also affects the self-assembly of peptides on the surface of mica. To explore the effect of pH on the formation of peptide nanostructures, we selected the mixture of water and TFE (*v/v*, 1:1) as the dissolvent system, and then adjusted the pH of the peptide solution with HCl and NaOH.

**Figure 5:**
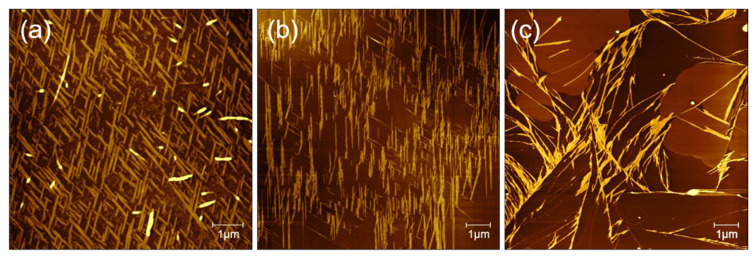
AFM images of self-assembled peptide nanostructures that formed on mica surface under different pH value: (**a**) pH = 2, (**b**) pH = 7, and (**c**) pH = 12.

Figure 5 presents typical AFM height images of peptide nanostructures that formed under different pH conditions on mica surface. In the context of pH = 2, peptides form a dense nanoribbon “cross-network” structure on the surface of mica. The reason for the formation of this dense and uniform “cross-network” nanobelt cluster is that the decrease in the pH of the solution regulates the adsorption of peptide molecules to mica and its incubation rate [31]. It is clear that the formed nanoribbons exhibit uniform orientations with a connection angle of about 60 degrees between the two directions (Figure 5a). We suggest that the GNNQQNY peptide has a whole isoelectric point of 5.52, which reveals positive charge in the acidic environment at pH = 2 [29]. Therefore, the electrostatic interactions between positively charged peptides and negatively charged mica surface mediated the surface structure-guided self-assembly of peptide molecules. When the solution pH is below 2, the number of peptide nanostructures becomes smaller and smaller, and when pH is below 1.6, there is virtually no orderly peptide nanoribbons on the surface of mica. An over-acid environment is not conducive to the incubation of peptide molecules on the surface of mica, which can lead to inactivation of peptide molecules.

Under neutral conditions, the number of peptide nanoribbons decreased and more nanofibers formed on the surface, as shown in Figure 5b. In addition, the density of the formed peptide nanostructures on the surface decreased significantly compared to those formed under acidic condition, and the formed peptide nanoribbons are arranged mainly in one direction. In alkaline environment (pH = 12), the formed nanofibers/nanoribbons become completely disordered (Figure 5c), and 2D large peptide layers appear. Here, it should be noted that the peptide layers are not formed on the surface of mica, as the alkaline environment facilitates the rapid assembly of peptides in solutions. On the other hand, peptides in alkaline environments are negatively charged, and negatively charged mica surface have electrostatic repulsion to the peptides, and therefore it is hard to induce the ordered self-assembly and arrangement of peptides on mica surface. These results reinforce the evidence that the formation of orderly peptide nanofibers/nanoribbons on mica is related to the electrical properties of both peptide and material interfaces. 

### 3.4. The Effect of Evaporation Period

The self-assembly of GNNQQNY peptides on mica surface is also related to the process of deposition and evaporation, in which the evaporation period of peptide solution plays an important role in the formation of final structures. The evaporation time of peptide solution on mica is actually affected by a variety of factors, such as the solvent type, air temperature, humidity, and flow conditions. To test the effect of the evaporation period on the self-assembly of GNNQQNY peptides on mica surface, we adjusted the flow of air to control the evaporation period of the peptide solution. 

**Figure 6:**
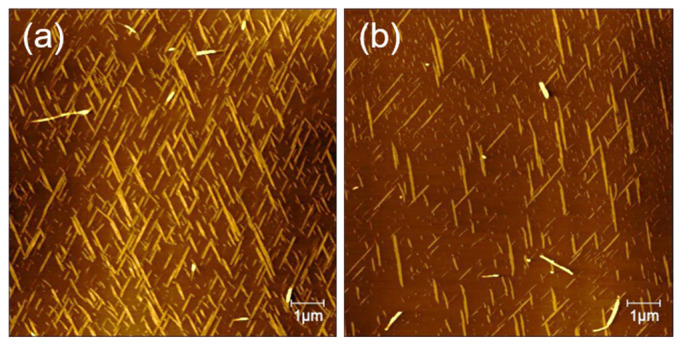
The effect of evaporation time on the formation of peptide nanobelts: (**a**) in a closed sample box and (**b**) under open room temperature environment.

Figure 6 show the AFM images of the peptide solution of mixture (*v/v*, 1:1) of TFA (0.1%) and TFE in the sample box and the open environment, respectively. In the first case, long evaporation time of peptide solution was set in a sample box, which provides enough molecular interactions and self-assembly for the formation of dense bi-directional nanoribbon arrays (Figure 6a). However, when the evaporation process was set in an open environment, it is hard to form bi-directional peptide arrangement but only one-directional peptide nanoribbons are found (Figure 6b). Based on the obtained AFM data, it can be concluded that the longer the evaporation time, the more peptides can promote the orderly growth of peptides on the surface of mica into dense bi-directional nanobelts structure.

### 3.5. Self-Assembly of Peptide on HOPG

**Figure 7:**
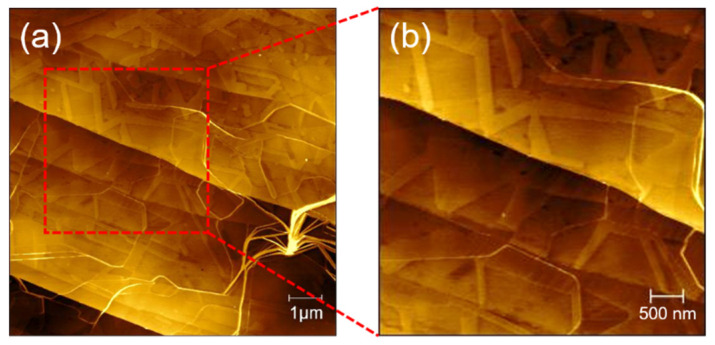
AFM images of self-assembled peptide nanostructures on HOPG surface: (**a**) 10 × 10 μm; (**b**) 4 × 4 μm.

In order to study the influence of different material interfaces on peptide self-assembly, we further examined the formation of peptide nanostructures on HOPG. Figure 7 shows the AFM image of a peptide solution of TFA (0.1%) and TFE mixture (*v/v*, 1:1) incubated on the surface of HOPG. As shown in Figure 7a,b, the surface of HOPG has a clear nanoribbon structure. The nanoribbons are arranged in two or even three directions (with an angle of 60 degrees). The height of the nanoribbons is about 1 nm, and the width is generally more than 200 nm. In the image, there are not only oriented nanoribbons, but also a certain number of curved nanofibers quickly assembled in the solution. Their height is higher than the self-assembled fibers on the surface, but they have no directionality. Compared with the incubation of peptides on the surface of mica under the same conditions, self-assembly on HOPG will obviously generate nanoribbons with a larger width and that are flatter. The distribution is not so dense, but it is easy to form nanoribbons in three directions. The differentiation of the self-assembly of GNNQQNY on the surface of mica and HOPG is due to the hydrophilic and hydrophobic properties of mica and HOPG and the difference in surface crystal structure.

### 3.6. Self-Assembly Mechanisms of Peptide on Mica and HOPG

The isoelectric point of the GNNQQNY peptide is 5.52, and under the condition of pH = 2, the positively charged peptide is organized and self-assembled on the negatively charged mica surface under the influence of electrostatic interaction. In previous studies, many peptides have been shown to form similar orderly structures on mica or HOPG, such as YSATSTY [27],QQKFQFQFEQQ [32],GAV-9 [26,33,34], and EAK16-II [35].

**Figure 8:**
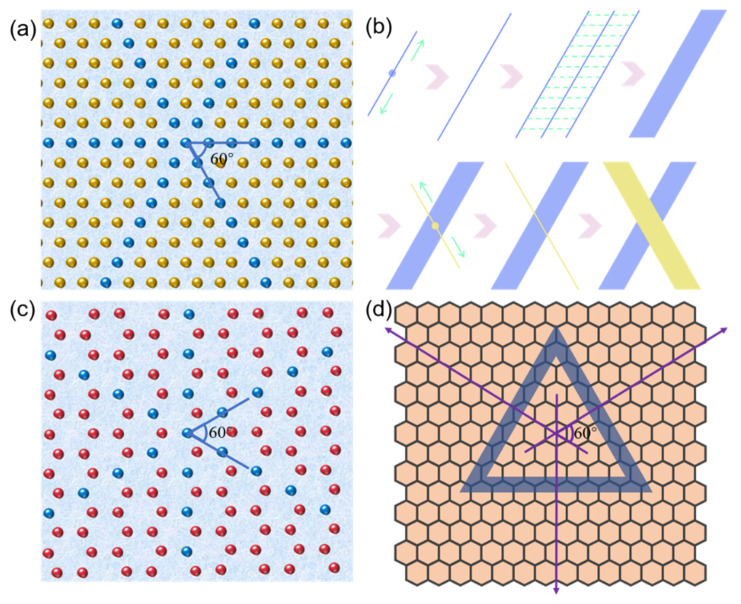
Self-assembly mechanisms of peptide on mica and HOPG: (**a**) hexagonal atomic arrangement of mica surface (the yellow balls represent the oxygen atoms on the mica, and the blue balls represent the oxygen atoms under the orderly coverage of the peptide.), (**b**) schematic self-assembly of peptide on mica surface, (**c**) hexagonal atomic arrangement of HOPG surface (the red balls represent the carbon atoms on the mica, and the blue balls represent the carbon atoms under the orderly coverage of the peptide.), and (**d**) schematic self-assembly of peptide on HOPG surface.

Based on our obtained results and previous reports, we proposed the self-assembly mechanisms of GNNQQNY peptide on the surface of mica and HOPG, as shown in Figure 8. When the peptide solution had just been dripped onto the surface of the clean mica, the peptide molecules in the solution were mainly present in a monomer state. As shown in Figure 8a, the surface of the freshly uncovered mica exposes the oxygen atomic layer of silicate, while other atomic layers of silicon, aluminum, and potassium are under it. As shown in Figure 8b, under the influence of the pseudo-hexagonal surface geometry template of mica (001), the polypeptide extension mica surface lattice began to spontaneously extend two-way in 2D layer, gradually forming a 1D nanofiber structure that arranged in a single direction. Among them, the structure formed by GNNQQNY peptides in this process is β-folding configuration. The 1D nanofiber structure is then further connected horizontally to form a 2D nanoribbon nanostructure. The self-assembly of peptides on mica templates tends to form single-directional 1D nanofibers and then horizontally connect to form 2D nanoribbons. When the self-assembly of peptides in a single direction reaches a certain saturation state, the peptides begin to repeatedly form nanofibers and connect horizontally into nanoribbons at an angle of 60 degrees in the previous direction. Therefore, in previous AFM images, we have observed both one-directional and bi-directional nanoribbons.

HOPG surface can be regarded as a single layer graphene structure, compared with the pseudo-hexagonal surface geometry template of mica (Figure 8c). However, HOPG surface hexagonal arrangement distribution is more obvious, so it is more conducive to the formation of peptide nanoribbons in three directions. Figure 8d presents a self-assembly mechanism of the GNNQQNY peptide on the HOPG surface. The self-assembly of peptides on HOPG can extend its lattice to form good nanoribbons. Since the HOPG terrace is flatter, the nanoribbons can be spread better and exhibit a higher width distribution, which has been identified by the AFM images shown in Figure 7.

## 4. Conclusions

In summary, we achieved the self-assembly of GNNQQNY peptide on the surface of mica and HOPG into an ordered nanoribbon or nanowire structures with an angle of 60 degrees by regulating the self-assembly conditions. Various influencing factors, such as the type of solvent, peptide concentration, pH value, and evaporation time, on the formation of peptide nanowires and nanoribbons with unidirectional and bidirectional arrays have been studied, and by adjusting and optimizing experimental conditions, it can effectively create ordered peptide nanobelts on mica and HOPG. Based on the above results, the self-assembly and formation mechanisms of peptide nanoribbons at the interface of the two materials are discussed. That is, the peptides are affected by the crystal lattice of the substrate and undergo 1D orderly epitaxial growth on the surface, and further they are laterally connected to form a nanometer belt. Our work explored the ordered self-assembly of specific peptides on the surface of materials, and it is beneficial to design more peptide sequences with high binding affinity to materials interfaces for the synthesis of functional hybrid bionanomaterials.

## Data Availability

Not applicable.
